# Expression of Concern: HCAP: Hybrid cyber attack prediction model for securing healthcare applications

**DOI:** 10.1371/journal.pone.0342838

**Published:** 2026-02-13

**Authors:** The PLOS One Editors

After this article [1] was published, it was noted that article uses terminology that differs from standards in the field (e.g., ‘cyber assault’ instead of ‘cyber attack’). In addition, the *PLOS One* Editors have concerns about the article’s compliance with the PLOS Authorship policy.

In light of the cumulative concerns, the *PLOS One* Editors issue this Expression of Concern. Readers are advised to interpret the article [1] with caution.
