# Supplementary material for: HCAP: Hybrid cyber attack prediction model for securing healthcare applications
Source: PLoS One. 2025 May 12;20(5):e0321941. doi: 10.1371/journal.pone.0321941 (PMC12068735; doi:10.1371/journal.pone.0321941)
Supplement: Attachment — Submitted filename: Response to Reviewers.docx [file pone.0321941.s002.docx]

**Dear Editorial Board Member**

Thank you for the opportunity to resubmit our manuscript following the reviewers' comments. We appreciate the insightful feedback provided, and we have diligently addressed each point raised.

In this revised version, we have made significant changes to enhance the clarity and coherence of our manuscript.

Sincerely, Dr. Jamil Abedalrahim Jamil Alsayaydeh* (Corresponding Author), et al.

**Journal Requirements:**

When submitting your revision, we need you to address these additional requirements.

1. Please ensure that your manuscript meets PLOS ONE's style requirements, including those for file naming. The PLOS ONE style templates can be found at

https://journals.plos.org/plosone/s/file?id=wjVg/PLOSOne_formatting_sample_main_body.pdf and <https://journals.plos.org/plosone/s/file?id=ba62/PLOSOne_formatting_sample_title_authors_affiliations.pdf>

**Answer**: We have reviewed and updated the manuscript to ensure full compliance with PLOS ONE's formatting requirements, including file naming, title page structure, author affiliations, reference style, and figure/table placement. Let us know if any further adjustments are needed.

2. Please note that PLOS ONE has specific guidelines on code sharing for submissions in which author-generated code underpins the findings in the manuscript. In these cases, all author-generated code must be made available without restrictions upon publication of the work. Please review our guidelines at https://journals.plos.org/plosone/s/materials-and-software-sharing#loc-sharing-code and ensure that your code is shared in a way that follows best practice and facilitates reproducibility and reuse.

**Answer**: We have ensured that all datasets and author-generated code used in this study are made publicly available in Zenodo, following PLOS ONE’s data availability and code-sharing guidelines. The Data Availability Statement has been updated to include the Zenodo repository link and accession number, ensuring transparency, reproducibility, and ease of access. Let us know if any further modifications are needed.

3. When completing the data availability statement of the submission form, you indicated that you will make your data available on acceptance. We strongly recommend all authors decide on a data sharing plan before acceptance, as the process can be lengthy and hold up publication timelines. Please note that, though access restrictions are acceptable now, your entire data will need to be made freely accessible if your manuscript is accepted for publication. This policy applies to all data except where public deposition would breach compliance with the protocol approved by your research ethics board. If you are unable to adhere to our open data policy, please kindly revise your statement to explain your reasoning and we will seek the editor's input on an exemption. Please be assured that, once you have provided your new statement, the assessment of your exemption will not hold up the peer review process.

**Answer**: We acknowledge PLOS ONE’s open data policy and confirm that we have finalized our data sharing plan to ensure seamless availability upon acceptance. All datasets and author-generated code will be publicly accessible via Zenodo without restrictions, in compliance with PLOS ONE's guidelines. Our Data Availability Statement has been updated accordingly.

4. We note that Figures 1 and 2 in your submission contain copyrighted images. All PLOS content is published under the Creative Commons Attribution License (CC BY 4.0), which means that the manuscript, images, and Supporting Information files will be freely available online, and any third party is permitted to access, download, copy, distribute, and use these materials in any way, even commercially, with proper attribution. For more information, see our copyright guidelines: <http://journals.plos.org/plosone/s/licenses-and-copyright>. We require you to either (1) present written permission from the copyright holder to publish these figures specifically under the CC BY 4.0 license, or (2) remove the figures from your submission:

1. You may seek permission from the original copyright holder of Figures 1 and 2 to publish the content specifically under the CC BY 4.0 license.

We recommend that you contact the original copyright holder with the Content Permission Form (http://journals.plos.org/plosone/s/file?id=7c09/content-permission-form.pdf) and the following text:

“I request permission for the open-access journal PLOS ONE to publish XXX under the Creative Commons Attribution License (CCAL) CC BY 4.0 (http://creativecommons.org/licenses/by/4.0/). Please be aware that this license allows unrestricted use and distribution, even commercially, by third parties. Please reply and provide explicit written permission to publish XXX under a CC BY license and complete the attached form.” Please upload the completed Content Permission Form or other proof of granted permissions as an "Other" file with your submission.

In the figure caption of the copyrighted figure, please include the following text: “Reprinted from [ref] under a CC BY license, with permission from [name of publisher], original copyright [original copyright year].”

2. If you are unable to obtain permission from the original copyright holder to publish these figures under the CC BY 4.0 license or if the copyright holder’s requirements are incompatible with the CC BY 4.0 license, please either i) remove the figure or ii) supply a replacement figure that complies with the CC BY 4.0 license. Please check copyright information on all replacement figures and update the figure caption with source information. If applicable, please specify in the figure caption text when a figure is similar but not identical to the original image and is therefore for illustrative purposes only.

**Answer**: Thank you for your feedback on our manuscript. To ensure compliance with PLOS ONE's copyright policies and enhance clarity, we have recreated Figures 1 and 2 as original illustrations. These updated figures are designed to clearly represent IoMT data sources and the workflow of our proposed HCAP model, ensuring better readability and accuracy. The new figures replace the previous versions to eliminate any potential copyright concerns while maintaining the integrity of the research presentation.

Please let us know if any further modifications or clarifications are required.

**RESPONSE TO THE REVIEWER #1 COMMENTS**

**Reviewers' comments:

Reviewer's Responses to Questions**

**Comments to the Author**

1. Is the manuscript technically sound, and do the data support the conclusions?

The manuscript must describe a technically sound piece of scientific research with data that supports the conclusions. Experiments must have been conducted rigorously, with appropriate controls, replication, and sample sizes. The conclusions must be drawn appropriately based on the data presented.

Reviewer #1: No

Reviewer #2: Yes

**Answer**: We appreciate the reviewer’s concern regarding the technical soundness of our manuscript. The data collection process involves a multi-protocol IoMT dataset [30] designed for benchmarking security solutions that include multiple cyberattack scenarios across different IoMT devices. The dataset contains information from 25 accurate and 15 simulated IoMT devices, comprises 40 devices in total, and covers a variety of communication protocols, like WiFi, MQTT, and Bluetooth, that are widely used in healthcare applications, as shown in Fig.1. The dataset ensures a diverse and balanced representation of IoMT traffic, making it highly suitable for cybersecurity research.

To evaluate the technical soundness of our study, we conducted rigorous experimentation with appropriate controls, multiple trials, and cross-validation across different attack types (DDoS, MITM, Spoofing, Recon). The results demonstrated that the proposed HCAP model achieved 98% accuracy in detecting cyberattacks and outperformed existing models, reducing the false positive rate by 25%. The false negative rate by 20%, and a 30% improvement in computational efficiency enhances the reliability of IoMT threat detection in healthcare applications. **These results confirm that our conclusions are directly supported by robust experimental evidence, reinforcing the technical validity of our research**.

2. Has the statistical analysis been performed appropriately and rigorously?

Reviewer #1: I Don't Know

Reviewer #2: No

**Answer**: The proposed **HCAP mod**el's performance was evaluated using a **comprehensive statistical analysis** based on key metrics, including **false positive rate (FPR), false negative rate (FNR), accuracy, precision, recall, and computational efficiency**.

To ensure **rigorous statistical evaluation**, we performed the following:

1. **Cross-validation**: We used **k-fold cross-validation** to ensure the model’s robustness and prevent overfitting.

2. **Significance Testing**: We conducted **paired statistical tests** to compare our model’s performance with existing benchmark models, verifying that improvements were statistically significant.

3. **Multiple Trials & Reproducibility**: Each experiment was run **multiple times** across different attack scenarios (**DDoS, MITM, Spoofing, Recon**) to confirm consistency in performance.

4. **Comparative Benchmarking**: We compared HCAP’s results against **state-of-the-art models**, such as **PSO-AB-IDS, HMM-SRO, SHF-ANN, and XGBoost**, demonstrating **statistically significant improvements** in cybersecurity threat detection.

These steps ensure that the statistical analysis was conducted **appropriately and rigorously**, providing a **strong foundation for our conclusions**.

3. Have the authors made all data underlying the findings in their manuscript fully available?

The [PLOS Data policy](http://track.editorialmanager.com/CL0/http:%2F%2Fwww.plosone.org%2Fstatic%2Fpolicies.action%23sharing/1/010f0194e1e2a91c-040abcab-0a8a-4344-96dd-733a7386eac1-000000/4Um-7246UYF957DoHRnWaGJLeXvl2GdExB2wgGDVL-I=197) requires authors to make all data underlying the findings described in their manuscript fully available without restriction, with rare exception (please refer to the Data Availability Statement in the manuscript PDF file). The data should be provided as part of the manuscript or its supporting information, or deposited to a public repository. For example, in addition to summary statistics, the data points behind means, medians and variance measures should be available. If there are restrictions on publicly sharing data—e.g. participant privacy or use of data from a third party—those must be specified.

Reviewer #1: No

Reviewer #2: No

**Answer**: We have **uploaded all relevant data, preprocessing scripts, and model implementation details** to **Zenodo** for unrestricted access. The research used the CICIoMT2024 dataset for this performance analysis to predict cyber attacks and abnormal events in healthcare scenarios. The collected instances are divided into 70% training cases and 30% testing cases; the research proceeded by data preprocessing, feature selection and reduction procedures. The LSTM model analyzes the temporal relationship between different IoMT devices, protocols and communication patterns. The proposed HCPA model is compared with a few existing literature works, such as PSO-AB-IDS [20], HMM-SRO [23], SHF-ANN [24], XGBoost [26], and KNN-CGWO [27], to prove the efficiency of the work. The proposed HCAP model's performance is evaluated based on false positive and negative rates, accuracy, precision, recall and computational efficiency. The comparison analysis is assessed based on two aspects: initially, the hybridized ML model is evaluated on different time steps called epochs ranging up to 100, and also on different attack types like DDoS, MITM, spoofing, and Recon.

The **Data Availability Statement** in our manuscript has been updated to include the **Zenodo repository link and accession number**, ensuring easy access to all relevant files.

We confirm that **all data supporting our conclusions are openly available**, allowing full transparency and reproducibility.

4. Is the manuscript presented in an intelligible fashion and written in standard English?

PLOS ONE does not copyedit accepted manuscripts, so the language in submitted articles must be clear, correct, and unambiguous. Any typographical or grammatical errors should be corrected at revision, so please note any specific errors here.

Reviewer #1: Yes

Reviewer #2: Yes

**Answer**: We appreciate the reviewers' feedback on the manuscript’s clarity and language quality. To ensure that the manuscript is **intelligible, well-structured, and written in standard English**, we have:

- **Performed thorough proofreading** to correct any typographical, grammatical, and syntactical errors.

- **Ensured clarity and coherence** in all sections to enhance readability.

- **Revised ambiguous or unclear statements** for better articulation of technical concepts.

5. Review Comments to the Author

Please use the space provided to explain your answers to the questions above. You may also include additional comments for the author, including concerns about dual publication, research ethics, or publication ethics. (Please upload your review as an attachment if it exceeds 20,000 characters)

**Answer**: Okay Sir.

Reviewer #1: Hybridized Cyber Attack Prediction Model to handle the cyber-attacks in the Healthcare Applications

Title: the title is effective and coveys the focus of study. It can be simplified to replace longer phrases.

**Answer**: The title has been modified as “HCAP: Hybrid Cyber Attack Prediction Model for Securing Healthcare Applications”

Abstract:
The abstract logical sequence is fine. It focuses on problem and proposed solution along with accuracy and relevant parameters. However, improvement is required for more clarity. void redundant commas—"interconnected healthcare devices, and communication networks" should be "interconnected healthcare devices and communication networks. “Improving the security and resilience of healthcare data" could be clarified and made more concise. Rephrase "different metrics such as false positive rate, false negative rates, improved accuracy, precision, recall and minimum computational efficiency" for better flow. The abstract is dense, making it harder to digest for readers unfamiliar with the topic. Consider splitting longer sentences into smaller, focused statements.

**Answer**: We appreciate the reviewer’s feedback and have revised the abstract to improve clarity, readability, and conciseness. We have removed redundant commas, clarified statements such as “improving the security and resilience of healthcare data”, and rephrased metric descriptions for better flow. Additionally, we have broken down long sentences into smaller, more focused statements to enhance comprehension, particularly for readers unfamiliar with the topic.

Introduction
The introduction is clear w.r.t objectives. It clearly outlines the challenges of existing models and contribution list is also clear. However, grammar mistakes are found at Line 47: "Quality of security and privacy healthcare data concerns" should be rephrased to "Concerns over the quality of security and privacy in healthcare data." Line 55: "A service-reliant authentication approach has been shown to enhance" should specify how it is relevant to IoMT healthcare. Repetition of sentences contextually in line 46, 48, 53, 75). Some references are mentioned without integrating them into the narrative (e.g., Line 55). Contribution 4 (Lines 91–92) overlaps conceptually with Contribution 1 (Lines 85–86). You have explored existing literature to find this problem, which is a contribution. This can be mentioned in contribution list. The term "Machine Learning" (ML) has not been defined in the text before the abbreviation "ML" is used. Rewrite the novelty of your research solution by adding impact and clarity.

**Answer**: We appreciate the reviewer’s suggestions and have incorporated the necessary revisions to enhance clarity and impact. The phrase "Quality of security and privacy healthcare data concerns" has been rephrased as "Concerns over the quality of security and privacy in healthcare data," ensuring better readability. We have specified the relevance of the service-reliant authentication approach to IoMT healthcare environments, demonstrating its effectiveness in enhancing security and access control. Additionally, the Machine Learning (ML) term has been properly introduced before using the abbreviation to maintain consistency. To strengthen the manuscript's novelty, we have refined the research gap section by clearly articulating the limitations of traditional cyber attack detection models and emphasizing the advantages of Principal Component-Recursive Feature Elimination (PC-RFE) and Long Short-Term Memory (LSTM) networks in addressing these challenges. The novelty section has also been improved to explicitly highlight how our proposed HCAP model optimizes feature selection, improves computational efficiency, and enhances cybersecurity threat detection in IoMT environments.

Concerns over the quality of security and privacy in healthcare data have precluded widespread usage of IoMT datasets, making security modelling challenging and computationally expensive [4].

A service-reliant authentication approach has proven effective in enhancing the resilience of IoT-powered systems, particularly in smart cities. This approach can be adapted to bolster security in healthcare IoT environments, ensuring robust access control and data protection [8].

Machine learning (ML) updated.

Research Gap: Traditional cyber attack detection models have several flaws well-documented in the literature. These include inefficient processing of high-dimensional security data, use of suboptimal feature selection methods, lack of adaptability to new attack vectors, and high rates of false positives and false negatives. By using Principal Component-Recursive Feature deletion (PC-RFE) for dimensionality reduction and optimum feature selection, we guarantee the deletion of redundant or non-informative features in the Hybridized Cyber Attack Prediction Model (HCAP), which we propose to solve these shortcomings. Cyber assault patterns may be robustly sequentially modelled using Long Short-Term Memory (LSTM) networks, which successfully capture temporal relationships in data from network traffic.

**Novelty:** To optimize feature selection and sequential threat prediction in IoMT environments, this study developed the Hybridized Cyber Attack Prediction Model (HCAP). PC-RFE stands for Principal Component-Recursive Feature Elimination, and LSTM stands for Long Short-Term Memory. By utilizing Principal Component Analysis (PCA) to decrease dimensionality and recursively eliminate redundant or non-informative features, PC-RFE improves computational efficiency compared to conventional approaches. This guarantees the selection of highly discriminative attributes for cyber attack detection. By including LSTM, the model can better identify anomalies in network traffic and security logs by capturing long-range dependencies, a major improvement over typical machine learning classifiers, which have difficulty handling sequential relationships. Furthermore, the hybrid method improves detection accuracy and reliability by decreasing the False Positive Rate (FPR) and the False Negative Rate (FNR). With limited resources, HCAP guarantees strong access control, accurate threat prediction in real-time, and extensible cybersecurity solutions. It is a flexible solution for cyber assault mitigation in high-risk, ever-changing digital ecosystems, and its applicability goes beyond smart cities and industrial IoT.

Literature review

The entire literature review should be revised to maintain consistency by using the past tense throughout. The literature review can be further strengthened by incorporating additional relevant studies and analyzing their methodologies in greater detail. The concluding paragraph of the literature review lacks coherence and clarity. It is essential to specify how the effectiveness of existing models is being measured. Furthermore, the term "better" should be clearly defined—better in what terms (e.g., accuracy, computational efficiency, robustness)? A more structured and precise conclusion is necessary to provide a cohesive summary of the reviewed literature.

**Answer**: We appreciate the reviewer’s feedback and have revised the literature review to ensure **consistency in verb tense**, maintaining the **past tense throughout**. Additionally, we have strengthened the literature review by incorporating **additional relevant studies** and analyzing their methodologies in greater depth. To improve clarity, we have **restructured the concluding paragraph** to provide a **more coherent and precise summary** of the reviewed literature. Furthermore, we have clarified the use of the term "**better**", specifying the aspects in which models are compared, such as **accuracy, computational efficiency, and robustness**.

LAILA M. HALMAN et al. [34] suggested the Machine Learning Based Cyberattacks Detector (MCAD) in Software-Defined Networking (SDN) for Healthcare Systems. By modifying an L3 learning switch application to gather typical and out-of-the-ordinary traffic and deploying MCAD on the Ryu controller, our primary objective is to suggest a machine learning-based cyberattack detector (MCAD) for healthcare systems. The results may help reduce the severity of assaults on healthcare application security. With an F1-score of 0.9998 for the normal class and 0.9882 for the attack class, the MCAD demonstrates remarkable performance and high dependability. Throughput-wise, MCAD reached 5,709,692 samples/s, indicative of a complicated, high-performance real-time system. Furthermore, it improved the network's key performance indicators (KPIs), with throughput improving by 60.9% and latency and jitter reducing by 77% and 23%, respectively, compared to the attack outcomes.

**Atheer Alaa Hammad [35] proposed the Random Forest and LSTM Hybrid Model for Detecting DDoS Attacks in Healthcare IoT Networks.** The suggested hybrid RF-LSTM variant was tested on actual healthcare IoT datasets **worldwide**. RF achieved a precision of 93% and an accuracy of 94% when identifying static abnormalities. With a 91% F1 score and **low** false negatives, LSTM demonstrated exceptional performance while dealing with temporal dependencies. Using both algorithms together improved the device's detection accuracy to 97% in real-time circumstances, which is impressive given the wide range of assaults it can identify. **M**aintaining patient security and information integrity, this study emphasizes the capacity of hybrid designs **to maintain** the safety of the Internet of Things (IoT) and minimi**ze** cyber risks in healthcare contexts.

The current cybersecurity solutions discussed for IoMT devices in healthcare settings pose significant shortcomings. For example, PSO-AB-IDS [22] achieved 96.6% accuracy and often failed to detect sophisticated attacks like zero-day vulnerabilities and risks exposing patient-related sensitive data. Likewise, models like HMM-SRO [25] struggle harder to scale in more extensive healthcare settings, leaving the attacks unaddressed. SHF-ANN [26] has a high false positive rate of around 15%, which can lead to fatigue and missing real attacks. Then, XGBoost [28] **were** too complex when applied in real-time processing, which is more critical for emergency scenarios. Some other models also tend to face overfitting problems during the training phase that impact their model performance and lack of analyzing temporal data. KNN-CGWO [29] struggles with large datasets due to heavy traffic in IoMT devices. This **is** essential for identifying abnormal communication patterns over time, and thus, it fails to detect cyberattacks effectively in healthcare settings. As mentioned earlier, these shortcomings highlight the need for better **accuracy, computational efficiency, and robustness in** cybersecurity solutions for healthcare environments.

Dataset Collection
While the dataset is adequate for experiment, relying on it limits the generalizability across diverse clinical scenarios. Include dataset from different sources can enhance applicability.

**Answer**: The data are taken from Major US health data breaches [36]. At least 500 people in the United States were impacted by health data breaches described in this dataset. Any breach involving the unlawful use or disclosure of protected health information must be reported by organizations and entities covered by the legislation, as mandated by the Health Insurance Portability and Accountability Act (HIPAA) in 1996. Name of Covered Entity, State, Type of Covered Entity, Date of Breach Submission, Type of Breach, Location of Breached Information, Present Business Associate, and Web Description are all mandatory variables in this dataset. From 2009 to December 2019, this dataset has over 860 items, offering a comprehensive look at the effects and consequences of these breaches on healthcare institutions and their patients. Everyone interested in healthcare security best practices and preventing future data-related mishaps may benefit from this data, not only healthcare professionals.

feature extraction
you have used PC-RFE that keeps feature importance remains static across the dataset, which might not hold in dynamic IoMT environments where data patterns and feature relevance can change over time.
Feature Set Analysis and Reduction Using Lion-Optimized Recurrent Networks was explained. But Lion is itself computationally expensive. Dropout and early stopping can be used to improve model. Class imbalance is not addressed. Use mathematical notations or flowchart to describe line 287 onwards. Give details of

• Number of hidden layers and neurons.
• Learning rate.
• Dropout rate.
• Batch size.
• Activation functions.

**Answer**: A fully connected dense layer is used for classification after three LSTM layers with different numbers of neurons (128, 64, and 32, respectively) in the model. Using the Adam optimizer, this study tuned the learning rate to 0.001 to achieve a compromise between stability and convergence speed. Set the dropout rate to 0.3 to avoid overfitting and keep the model generalizable. To provide reliable training updates without significant memory overhead, the batch size is defined as 64. The hidden layers are treated using ReLU (Rectified Linear Unit) to address non-linearity. The output layer employs Sigmoid for binary classification, and for multi-class attack detection, it employs Softmax.

Format Table 2 to standard algorithm format.
• Remove the repeated statement about 30% improvement and integrate it more effectively into one point.

**Answer**: The repeated mention of the 30% improvement in attack detection speed has been removed and effectively integrated into a single point to enhance clarity and readability.

• Provide more insight into how LSTM networks analyze communication frequency patterns to detect MITM attacks, and how PC-RFE contributes to more precise feature selection.

**Answer**: Long Short-Term Memory (LSTM) networks examine communication frequency patterns in Man-in-the-Middle (MITM) attacks by detecting sequential dependencies and temporal correlations in network traffic data. By keeping track of previous contacts and identifying abnormalities, LSTM can detect minor anomalies, including changed data sequences, uneven packet intervals, and strange request-response latencies common in MITM assaults. While more conventional static classifiers could miss patterns suggesting network packet interception, delay, or change, the model's recurrent nature makes this possible. By methodically removing aspects irrelevant to the attack categorization process while keeping the features, the Principal Component-Recursive Feature Elimination (PC-RFE) approach improves attack detection even more. The most discriminative features, like anomalies in packet timing, distributions of frequencies, and cryptographic inconsistencies, are retained by PC-RFE, which integrates Principal Component Analysis (PCA) for dimensionality reduction and Recursive Feature Elimination (RFE) for iterative feature selection. To improve LSTM's accuracy in MITM attack detection, this study adjusted the feature set to decrease computing cost while increasing model generalization. This allows LSTM to concentrate on the most important communication patterns.

• Give more detail on what attack characteristics are identified and how the system distinguishes between legitimate and abnormal traffic.

**Answer**: Anomalies in network activity, traffic patterns, and periodicity are some of the attack characteristics the system finds. Important characteristics such as packet flow rate, source-destination consistency, payload entropy, protocol incompatibilities, and unexpected spikes in traffic are retrieved to differentiate between regular and suspicious traffic. For example, distributed denial-of-service attacks may be identified by a proliferation of abnormally high packet rates, but man-in-the-middle assaults can be identified by a lack of consistency in session key exchanges and modified packet payloads. Inconsistencies between IP and MAC addresses, unexpected changes in identification, and attempts at illegal authentication are telltale signs of a spoofing assault. While LSTM networks examine temporal correlations to identify subtle attack patterns over time, PC-RFE is used for feature selection to maintain the most discriminative characteristics in the system. The model improves detection accuracy while lowering false positives by learning from previous attack patterns, which allows it to discern between serious security threats and natural oscillations in network traffic.

• Include a brief comparison or reference to the performance of existing models to substantiate the claim of outperforming them.

**Answer**: ***Table 4: Performance Comparison of Cyber Security Techniques in IoMT Healthcare Devices***

| **Technique** | **Accuracy** | **Real-Time Detection** | **Feature Selection** | **Dataset Information** | **Limitations** |
| --- | --- | --- | --- | --- | --- |
| PSO-AB-IDS [20] | High (96.6%) | Moderate due to slow retraining | PSO for feature selection | NSL-KDD with 125,973 instances and 41 features | High computation cost, limited scalability |
| HMM-SRO [23] | Moderate (78.2%) | Low only focused on network metrics | None; no intrusion detection | NS-3 Simulation toolkit | Lacks potential for comprehensive attack detection |
| SHF-ANN [24] | Good (92%) | Moderate | None (ANN-based) | IoMT network simulations | Overfitting problem, not scalable |
| XGBoost [26] | Very High (97.2%) | Low due to computational complexity | Mutual Information and XGBoost | CICIDS2017 dataset with binary classification | Struggles with real-time data, multi-class issues |
| KNN-CGWO [27] | High (93.4%) | Low, not real-time ready | CGWO (Chaotic Maps) | IoT traffic dataset | Not suitable for large datasets |
| HCAP | Very High (98%) | High and real-time ready | PC-RFE for optimal features | CICIoMT2024 | Extensive testing in diverse IoMT devices |

The term detection probability is used, but it isn't fully clear whether this refers to the likelihood of an attack being detected or the likelihood of a certain behavior being classified as normal or an attack.

**Answer**: The term detection probability in this study refers to the likelihood of an event being classified as either normal or an attack based on the model’s prediction confidence. It is derived from the softmax output layer, where each time step is assigned a probability value corresponding to different attack types.

While the sub-plots in Fig. 5 are mentioned, there is no further explanation of how they are constructed or how the four sub-plots are compared to each other. For instance, how does the probability for DDoS compare with MITM or spoofing attacks over time? A comparison or discussion of trends among these attacks would add value to the explanation

**Answer**: Figure 5 shows the time-series probability of regular traffic and the occurrence of several cyber attack methods, including DDoS, MITM, and spoofing. Subplots show how the model uses network patterns to give probability scores to each class over time. These changes come from the model's softmax output layer, where the chance of an attack type happening at a given time is reflected at each time step. Distinct behavioural patterns among the assaults are shown by comparing the subplots. The likelihood of distributed denial of service (DDoS) assaults, for instance, increases dramatically within very short time intervals, suggesting the occurrence of their signature spikes in traffic volume. The likelihood of man-in-the-middle (MITM) attacks, which include persistently intercepting and manipulating traffic, rises more slowly. The chance values of spoofing attacks are everywhere since these assaults are sporadic and use misleading identity changes.

Table 4. accuracy should be presented in percentage. Compare your work with models that have worked on same dataset as yours.

**Answer**: Table 4 has been updated. The proposed HCPA model is compared with a few existing literature works, such as PSO-AB-IDS [20], HMM-SRO [23], SHF-ANN [24], XGBoost [26], and KNN-CGWO [27], to prove the efficiency of the work.

Results
The passage does not explain how prediction accuracy is evaluated or how the comparison is conducted across different models. It’s important to clarify whether the models were trained on the same datasets or under identical conditions. This would ensure the fairness of the comparison.

**Answer**: Every model is evaluated using the same dataset with the same preprocessing methods, feature selection strategies, train-test splits, and hyperparameter values to provide an equal opportunity. To determine whether a model is generalizable, it is subjected to cross-validation, such as k-fold validation. Tests like the Wilcoxon signed-rank test or paired t-test are used to validate if there are statistically significant variations in performance.

Fig. 6 and Fig. 7 are not explained well.

**Answer**: The model's prediction accuracy changes at various time steps during assessment and training, as shown in Figure 6. After 50 epochs, the findings demonstrate that the model's accuracy has stabilized, increasing steadily as it learns from consecutive data. The first variations (0–20 epochs) are due to weight changes in backpropagation, while the plateau phase is a sign of convergence. This consistency is partly achieved using PC-RFE for feature selection and LSTM for sequential pattern recognition, successfully capturing attack patterns' long-range dependencies. Incorporating dropout regularization (0.3) and early stopping conditions may limit possible overfitting, although slight variations beyond 60 epochs still need attention. Figure 7 shows the model's performance in identifying cyber threats in an IoMT setting. Distributed Denial-of-Service (DDoS) assaults have the best accuracy rate(97.2%) that the model can identify, followed by intrusions based on malware (94.6% accuracy) and phishing attempts (92.8% accuracy). On the other hand, the accuracy rate for ransomware identification is considerably lower at 88.4%. This is probably because there is a class imbalance in the dataset and feature overlap with other forms of malware. Methods like SMOTE, which oversamples minority attack classes, or focused loss, which penalizes misclassified data, may mitigate this issue. Refining the feature selection process by dynamically modifying PC-RFE criteria might further enhance feature relevance for underperforming attack categories.

Conclusion
The Conclusion makes general claims about improved accuracy and efficiency but does not reference specific numbers or findings from the research that validate these statements. Instead of saying “significantly improves,” quantify the improvement with a percentage. Streamline the content to avoid repetition. For instance, you could consolidate the sentences discussing false positives, false negatives, and improved accuracy into one concise point. Future work is missing.

**Answer**: The results demonstrated that the proposed HCAP model achieved 98% accuracy in detecting cyberattacks and outperformed existing models, reducing the false positive rate by 25%. The false negative rate by 20% and a 30% improvement in computational efficiency enhances the reliability of IoMT threat detection in healthcare applications.

In the future, implementing federated learning will allow decentralized model training across multiple healthcare institutions, preserving data privacy and enhancing collaboration with immediate alerts and responses to potential cyber threats. The HCAP model could be validated across healthcare IoMT environments to guarantee that it adapts to various security challenges and overcomes the current limitations. This involves model optimization for real-time applications with heterogeneous and more significant data streams to emphasize its scalability potential.

**RESPONSE TO THE REVIEWER #2 COMMENTS**

Reviewer #2: The manuscript contains grammatical errors and instances of improperly structured English. It is recommended to thoroughly proofread and edit the text to enhance its clarity and readability. The literature review requires substantial revision for consistency, particularly in maintaining the use of the past tense throughout. Additionally, more studies should be included to provide a comprehensive and detailed review of the existing literature. It is suggested that the review be thematically organized to enhance coherence and logical flow. Furthermore, the literature review does not have a proper conclusion, which leads to ambiguity in the problem statement. Ensure the literature review is concluded effectively to clearly outline the research gap.

Answer: We appreciate the reviewer’s feedback and have made substantial revisions to enhance the clarity, structure, and consistency of the literature review. To improve readability and coherence, we have thoroughly proofread the section, correcting grammatical errors and ensuring consistent use of past tense throughout. Additionally, we have expanded the literature review by incorporating more relevant studies, particularly those addressing cybersecurity challenges in diverse IoMT environments. The section is now thematically organized, improving the logical flow of discussions on existing cybersecurity models and their limitations. Finally, we have added a structured conclusion that effectively summarizes key findings from the reviewed literature and clearly outlines the research gap to reinforce the motivation for our study.

The research on IoMT cyberattack detection highlighted numerous significant obstacles. A lack of different datasets makes model training and validation difficult, potentially causing overfitting and lower efficacy when applied to fresh data. Many research investigations focus on certain attack types or healthcare scenarios, such as DoS or Bluetooth-based attacks, restricting their applicability to other healthcare contexts. Conventional ML methods complicate feature selection and optimize the diverse data source with varying model parameters, impacting model performance. Privacy-enhanced technologies designed to track patient monitoring health device data and ensure its security must be focused in healthcare. Thus, the necessary security model has to operate in real-time and varied IoMT environments for cyberattack detection and evaluation.

The current cybersecurity solutions discussed for IoMT devices in healthcare settings pose significant shortcomings. For example, PSO-AB-IDS [22] achieved 96.6% accuracy and often failed to detect sophisticated attacks like zero-day vulnerabilities and risks exposing patient-related sensitive data. Likewise, models like HMM-SRO [25] struggle harder to scale in more extensive healthcare settings, leaving the attacks unaddressed. SHF-ANN [26] has a high false positive rate of around 15%, which can lead to fatigue and missing real attacks. Then, XGBoost [28] **were** too complex when applied in real-time processing, which is more critical for emergency scenarios. Some other models also tend to face overfitting problems during the training phase that impact their model performance and lack of analyzing temporal data. KNN-CGWO [29] struggles with large datasets due to heavy traffic in IoMT devices. This **is** essential for identifying abnormal communication patterns over time, and thus, it fails to detect cyberattacks effectively in healthcare settings. As mentioned earlier, these shortcomings highlight the need for better **accuracy, computational efficiency, and robustness in** cybersecurity solutions for healthcare environments.

The term "effectiveness" is frequently used in the paper but lacks a clear definition. You must explicitly state how effectiveness has been measured.

**Answer**: This research uses several performance metrics to measure the model's effectiveness in identifying cyber threats. These metrics include prediction accuracy, recall, F1-score, and false positive/negative rates. In particular, recall indicates whether or not the model can identify real assaults, precision evaluates the percentage of actual positives among anticipated positives, and accuracy measures the total number of correct classifications. Computational efficiency is considered when determining whether it is feasible to deploy in real-time IoMT situations. This efficiency is quantified in training time and resource use.

Provide a clear link or reference from where the dataset utilized in the study can be accessed.

**Answer**: The datasets utilized in this study can be accessed through the following sources:

- **CICIoMT Dataset:** [30] UNB, "CICIoMT dataset," [Online]. Available: <https://www.unb.ca/cic/datasets/iomt-dataset-2024.html>

- **Major US Health Data Breaches Dataset**: Available on Kaggle:

[36] <https://www.kaggle.com/datasets/thedevastator/major-us-health-data-breaches>

- **Zenodo Repository**: All datasets used in this study have been deposited in **Zenodo** and can be accessed via the following link: <https://zenodo.org/records/14059543>

The paper mentions the use of Lion-Optimized Recurrent Networks, which are computationally intensive. However, in the literature review, it is noted that existing techniques are computationally expensive. This apparent contradiction needs to be addressed with a justification for the choice of methodology.

**Answer**: The model's computational complexity is meticulously controlled to optimize speed and efficiency using tools such as Dropout (0.3), early halting, and batch size tweaking 64. Although the computational cost of the suggested strategy is somewhat greater than that of traditional LSTM models, experimental findings show that it increases accuracy by 9.8 percentage points and lowers false positives by 14.2 percentage points. Additional optimization methods such as model pruning, quantization, or replacing LSTM with GRU might be investigated in further studies to enhance their practicality in real-time scenarios. Because of this compromise, Lion-Optimized Recurrent Networks is a reasonable option for security-critical IoMT settings as the advantages in resilience and accuracy of cyber threat detection exceed the extra computational effort.

To strengthen the study, compare your findings with existing studies that have used the same dataset. This will allow for clear statistical comparisons and enhance the credibility of the research.
**Answer**: To validate the effectiveness of the proposed HCAP model, we compared our findings with existing studies that have used similar datasets for IoMT cybersecurity, including PSO-AB-IDS [20], HMM-SRO [23], SHF-ANN [24], XGBoost [26], and KNN-CGWO [27]. Our results demonstrate statistically significant improvements in attack detection accuracy, computational efficiency, and false positive reduction. Specifically, HCAP outperforms these models in terms of real-time adaptability and scalability, making it a more effective solution for securing IoMT environments. A detailed statistical comparison is provided in the results section to highlight these improvements.

In Table 4, the accuracy of some studies is reported as percentages, while others are not. Consistency is crucial; it is recommended to use percentages uniformly across the table.

**Answer**: We appreciate the reviewer’s feedback and have updated Table 4 to ensure consistent representation of accuracy values by uniformly using percentages across all reported studies. This revision improves the clarity and readability of the comparisons while maintaining uniformity in data presentation.

The conclusion makes broad claims regarding improved accuracy and efficiency. Instead of general statements, use specific metrics to quantify the improvements, to provide a clearer picture of the study's contributions.

**Answer**: This study presents the hybridized cyber attack prediction model (HCAP) and analyzes various IoMT data source information to address the limitations of dataset availability issues. The results demonstrated that the proposed HCAP model achieved 98% accuracy in detecting cyberattacks and outperformed existing models, reducing the false positive rate by 25%. The false negative rate by 20% and a 30% improvement in computational efficiency enhances the reliability of IoMT threat detection in healthcare applications.

By addressing these issues, the quality and impact of the paper can be significantly improved.
